# Effect of postmortem pH on the physical, biochemical, and sensory characteristics of bovine *longissimus thoracis et lumborum* muscle

**DOI:** 10.1002/fsn3.3235

**Published:** 2023-01-22

**Authors:** Yunus Khatri, Elisabeth Huff‐Lonergan

**Affiliations:** ^1^ Department of Food Science and Nutrition University of Leeds Leeds UK; ^2^ School of Science Engineering and Technology RMIT University Ho Chi Min City Vietnam; ^3^ Department of Animal Science Iowa State University Ames Iowa USA

**Keywords:** beef, DFD, palatability, pH, postmortem changes, SDS‐PAGE, tenderness

## Abstract

From a large feeding trial study consisting of 299 bulls and steers, 15 carcasses exhibited stress‐related syndromes manifested by atypical color and pH which were then selected for subsequent analysis. Samples of *longissimus thoracis et lumborum* muscle with postmortem pH in the range of 5.5–6.9 were subjected to a 14‐day aging period at 2°C. Sensory panel tenderness, connective tissue, juiciness, and flavor intensity of high pH (6.4–6.9) meat were significantly different (*p* < .05) from samples of intermediate pH (6.0–6.1) as well as normal pH (5.5). Muscles at pH 6.0–6.1 were the toughest samples. This was confirmed by Warner–Bratzler shear force (WBSF), residual force, and myofibril fragmentation index. Palatability attributes of normal pH (5.5) samples were significantly different (*p* < .05) from dark‐cutting beef in terms of tenderness and flavor and at the high pH extreme. The increase in WBSF at pH 6.0–6.1, lack of extensive degradation of muscle proteins, and the decreased sarcomere length resulted in tougher meat than low or high pH muscles. Sodium dodecyl‐sulfate polyacrylamide gel electrophoresis of meat at the high pH extreme (6.7–6.9) revealed that the breakdown of troponin‐T to 30 kD was complete while at intermediate pH (6.0–6.1) was incomplete. In addition, the appearance of a ‘doublet’ on high‐molecular‐weight resolution gels may also account for the greater tenderness experienced by sensory panelists.

## Graphical abstract

Protein degradation patterns of both high‐ and low‐molecular‐weight structural proteins are examined. The complete breakdown of both troponin‐T as well as the majority of the 30 kD component occurred at the high pH extreme. Intermediate pH revealed incomplete troponin‐T breakdown and this may be related, in part, to its toughness. In addition, the breakdown of troponin‐I in pale soft exudative beef may indicate different enzymatic mechanisms (e.g., catheptic breakdown) involved in proteolysis.

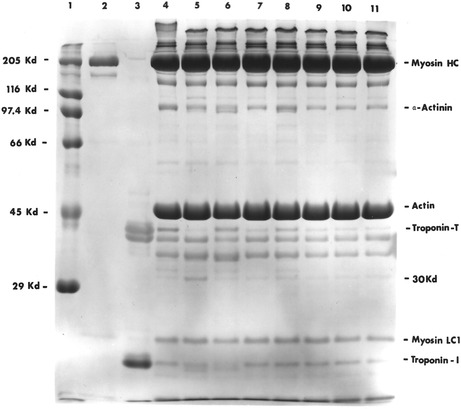

## 1 INTRODUCTION

The ultimate pH of beef carcasses is a widely used indicator of meat quality. However, the effects of stress contribute to the decline in pH postmortem and thus pre‐ and postslaughter care are important factors in determining the eating quality of meat. The complexity of meat tenderness has been shown to be related to breeding, diet, and handling (Bekhit et al., 2014); the structure, composition, and amount of intramuscular connective tissue (Nishimura, 2010), state of muscle contraction (Kerth, 2013), enzymatic action on myofibrillar/cytoskeletal proteins (Gagaoua et al., 2020; Huff‐Lonergan et al., 2010), postmortem temperature (Warner et al.,^2014^), and ultimate muscle pH (Gonzalez‐Rivas et al., 2020). Muscle fiber size and muscle fiber type (Lang et al., 2020) have also been implicated.

A comprehensive review of stress effects on meat quality is provided by Xing et al. (2019). Stress defined by Wiepkema and Koolhaas (1993) occurs when an animal is exposed to intrinsic or extrinsic stressors, which may disturb the normal physiological equilibrium, thereby eliciting a threatened homeostatic state. It leads to the depletion of glycogen in muscle affecting the ultimate pH following postmortem glycolysis. Stress may be manifested by a number of factors including sex, breed, animal diet, weather and season, transport and lairage holding times, psychological stress caused by changed environments, proximity and interaction with humans, and changes in herd hierarchy (Ponnampalum et al., 2017). Moreover, acute heat stress immediately prior to slaughter accelerates muscle glycogenolysis and lactic acid concentration and produces a rapid drop in muscle pH early postmortem while the carcass is still hot. Under these circumstances, the result is pale soft exudative (PSE) meat typically of low water‐holding capacity. In contrast, chronic heat stress experienced by animals leads to depleted reserves of muscle glycogen and thus reduced amounts of lactic acid production. The result of this condition is dark firm dry (DFD) meat with high water‐holding capacity (Gonzalez‐Rivas et al., 2020).

In the main, numerous studies have shown that meat from bulls has been rated as less tender than that from steers (Jeremiah et al., 1988; Knight et al., 2004; Reddy et al., 2015). Using young bulls between 11 and 15 months has held promise for greater acceptability in terms of tenderness and flavor. Nakitari's thesis (2021) investigated meat quality attributes of young bulls and steers and found no difference between bulls and steers for meat color, Warner–Bratzler shear force (WBSF), and myofibril fragmentation index (MFI), and due to aging not influencing meat tenderness (*p* = .682) recommended that they could be placed in one category in a classification scheme.

Postharvest meat processing practices, particularly aging, are well documented to play a pivotal role in the establishment of beef palatability. During aging, enzymes from the beef itself degrade the proteins, carbohydrates, and hexane (Hwang et al., 2022). However, pH has a profound impact on proteolytic enzyme activities, subsequently affecting the aging potential for meat tenderization. In a fairly recent review, Kim et al. (2018) state that specific conditions to maximize aging impacts on meat quality attributes have not been fully established. Thus, pH has been the focus of this study. When considering proteolysis, neutral/alkaline protease activity is said to occur while carcass temperatures and pH are high (Dayton et al., 1975; Marsh, 1983), or acidic cathepsins when pH is low (Dutson et al., 1980). Retention of calpain activity under acidic conditions has also been reported (Koohmaraie et al., 1986). However, postmortem pH and temperature are generally agreed to have a significant effect on final muscle tenderness (Marsh et al., 1981). Geesink (1993) reported decreased degradation of titin and increased degradation of nebulin postmortem in beef muscle with a low pH, compared with beef muscle of high pH.

The objective of the present study was to investigate the tenderness of beef with respect to sensory, physical, and biochemical parameters, using naturally occurring meat at varying pH. Although some reports have been published on the effects of dark cutting on tenderness, data addressing varying degrees of dark cutting across a large pH range is lacking. Furthermore, limited information exists regarding PSE beef.

## 2 MATERIALS AND METHODS

This study was approved by the Institutional Review Board of Iowa State University. Written informed consent was obtained from all study participants. Ethics Committee approval was received—number 09/1992.

As part of a large study, a total of 299 (*N* ~100 animals/farm) yearling bulls and steers were born and raised at three Iowa State University farms. The animals were of similar genetic makeup (Jersey × Angus cross) and environmental influence. Calves were fed concentrate and straw ad libitum after weaning at approximately 6 months. These animals at an age of 12–15 months were transported the day prior, a distance of 60–80 Km during the Summer period, to an approved commercial slaughter facility in Des Moines, Iowa, at three slaughter periods, around 30 days apart. Standard methods of slaughter and dressing practice were followed without electrical stimulation.

Carcasses were moved into a cold room at 4°C where they were held overnight. The following day each side was quartered between the 12th and 13th vertebrae. USDA yield and quality grades were determined after 24–30 h postmortem by three trained personnel from Iowa State University and one USDA Grader. Color measurements were also undertaken following exposure to 30 min of atmospheric oxygen. Samples of *longissimus thoracis et lumborum* (LTL) were excised from the 11th and 12th rib as described in Goñi et al. (2007), fabricated into two equal 2.5 cm portions from the right‐hand side of the carcass. The posterior end was used for chemical analysis, MFI, sodium dodecyl‐sulfate polyacrylamide gel electrophoresis (SDS‐PAGE), and sarcomere length determinations while the anterior portion was used for sensory and WBSF measurement. Each cut was individually wrapped, labeled, and shipped on ice under refrigerated conditions (4°C) to Iowa State University Meat Laboratory, accounting for approximately 60‐min drive.

This current work utilized a subset of 15 carcasses that exhibited stress‐related syndromes which were classified as dark cutting (DFD) pH ≥6.0 (*n* = 14) and pale soft and exudative (PSE) pH = 5.5 (*n* = 1). Data from color measurements were used to assist in the differentiation of the syndromes. From the same study, 15 control samples of similar sex (normal pH = 5.5) were also obtained.

### 2.1 Ultimate pH measurements

A Beckman ChemMate pH meter equipped with a surface probe was used to measure pH at a 24‐h postmortem. The pH meter was calibrated using standard buffers at pH 4.0 and 7.0. Based on the aging studies by Jiang et al. (2010) and Barón et al. (2021), no further pH measurements after 24 h were carried out. Kim et al. (2015) and Li et al. (2013) posited that over a duration of 2 weeks, aging had little or no impact on the pH regardless of the aging method.

### 2.2 Vacuum packaging and aging

Samples were vacuum packed (Dixiepac 100) in polyamide, polyethylene PA/PE‐70 bags (Cryovac) and aged at 2°C for 12.5 days prior to freezing at −18°C (Ren et al., 2021) for use as and when required over a duration of 2 months. The authors acknowledge that freezing does affect tenderness as mentioned in Grayson et al. (2014); however, this factor did not form part of our study. It is important to note that after pH measurement, preparation by fabrication, shipping, vacuum packaging, and labeling of nearly 100 samples per lot, approximately 12–14 h would have elapsed. The 12.5‐day vacuum packaged aging period was chosen due to the 24‐ to 30‐h aging on hook prior to grading and fabrication plus the further 14‐h preparation time, thus producing steaks aged 14 days from the time of slaughter. Colle et al. (2016) found that an aging period of 14 days was commonly used for optimizing beef eating quality, relating to a plateau in proteolysis tenderization with minor or little improvement in tenderness beyond such a period.

### 2.3 Color measurement

Steak surfaces were assessed using a handheld HunterLab MiniScan colorimeter (HunterLab) using the *L*, *a*, *b* system. The colorimeter was calibrated using a No. 2 HunterLab calibration plate (*L* = 67.5, *a* = +23.7, *b* = +12.5). Readings were then taken at three different surface locations for each muscle and the values were averaged.

The following analyses given below were conducted after a 14‐day aging period.

### 2.4 Sensory evaluation

Steaks were thawed for 48 h at 2°C and then broiled to an internal temperature of 70°C. The internal temperature of steaks was monitored at the geometric center using copper–constantan thermocouples attached to an Omega Digital Trendicator. Steaks were broiled to an internal temperature of 40°C, turned, and then broiled further to a final temperature of 70°C. Once removed from the broiler, steaks were individually wrapped in aluminum foil, placed in a 60°C warming oven, removed, and cut into approximately 1.27 × 1.27 × 3.18 cm sample cubes. Two samples randomly chosen were served to an 8‐ to 10‐member trained sensory panel. Panelists were individually seated in booths in a room separated from the preparation area and provided with water to rinse their palates between samples. Red fluorescent lights were used to eliminate any biases due to the color of the samples.

Panelists (seven males and three females aged between 22 and 43 years) were trained in accordance with the guidelines provided by the American Meat Science Association (2015). All individuals were beef eaters (at least four times a week) from the Department of Animal Science and Food Science and Nutrition. Participants although previously trained, received six 2‐h training sessions in a 2‐week period immediately preceding testing. During each training session, there was both discussion and sensory assessment of representative samples using the following sensory definitions: Myofibrillar tenderness—a measure of how easily the sample breaks down into smaller pieces after three chews; Connective tissue—the amount of insoluble connective tissue that remains in the mouth after thorough chewing; Juiciness—the sensation of free fluids released from the meat upon chewing; Flavor intensity—a measure of the perceived intensity of beef flavor; and Flavor—a measure of the sensation perceived as beef flavor. Scores for tenderness (T), connective tissue (CT), juiciness (J), flavor intensity (Fl), and flavor (F) were based on an 8‐point descriptive scale (tenderness 1 = extremely tough, 8 = extremely tender; connective tissue, 1 = abundant, 8 = none; juiciness, 1 = extremely dry, 8 = extremely juicy; flavor intensity, 1 = extremely bland, 8 = extremely intense; flavor, 1 = extremely off flavor, 8 = extremely flavorful). All participants were able to achieve repeatable evaluations and were thus selected for this analysis. Values for each sensory attribute of a steak were reported as an average of panelist scores.

### 2.5 WBSF measurements

Three cores of 1.3 cm diameter were removed parallel with the axis of the muscle fiber from each of three sections (central, medial, and lateral) of cooked steaks previously cooled to room temperature (25°C) and used for WBSF assessment. Shear force measurements were carried out using the WBSF attachment of the Instron Universal Testing Machine (Instron Corp.). A 50‐kg load cell and a cross‐head speed of 100 mm/min were used. Shear force deformation curves were recorded by means of a chart recorder using a chart speed of 100 mm/min. Each core was sheared twice along the long axis and the values were averaged. Moller (1981) reported stronger correlations with sensory panel data by dividing curves into two parts. The first yield point was identified as the compression/myofibrillar force corresponding to the myofibrillar component of tenderness, while the second yield point relates to the residual force of the connective tissue component of tenderness. Values for each steak are reported in kg/cm^2^.

### 2.6 Electrophoretic separation of myofibrillar proteins

Electrophoretic isolation of myofibrils from *longissimus thoracis* muscles was carried out according to the procedure described by Goll et al. (1974). The protein content of the myofibril preparation was determined using the modified method by Robson et al. (1968). SDS‐PAGE was performed in line with Laemmli (1970) using 5% polyacrylamide (acrylamide/bisacrylamide = 100:1 w/w) and 12% polyacrylamide (acrylamide/bisacrylamide = 37:1, w/w) slab gels (pH 8.9) of 16 cm × 14 cm × 0.15 cm dimension. The 12% gels were layered with 5% stacking gel. Protein samples were incubated at 50°C for 20 min in 0.5 ml tracking dye solution (30 mM Tris‐HCI, pH 8.00, 3 mM EDTA, 3% [w/v] SDS, 30% [v/v] glycerol, and 0.3% [w/v] pyronin Y) and 0.1 ml of 2‐mercaptoethanol (Wang, 1982). Gels were stained with Coomassie brilliant blue (0.2% [w/v] Coomassie brilliant blue R‐250, 7% 9 [v/v] acetic acid, 40% [v/v] ethanol). Standards used include myosin (205 K); β‐galactosidase (116 K); phosphorylase B (97.4 K), bovine albumin (66 K); egg albumin (45 K); and carbonic anhydrase (29 K) (supplied by Sigma Chemical Company).

### 2.7 MFI determination

Myofibril fragmentation index was determined on each steak by a modified procedure described by Parrish and DePulgar (1990). Three cores from each steak (medial, central, and lateral) were finely scissor‐minced, and readily apparent pieces of fat and connective tissue removed. Four grams of this sample was homogenized for 30 s in 10 volumes (w/v) of a 2°C isolating medium of 100 mM KCl, 20 mM K phosphate, 1 mM EGTA, 1 mM MgCl_2_, and 1 mM sodium azide. The homogenate was then passed through a polyethylene strainer to remove connective tissue and debris. A stirred amount (0.25 ml) of the homogenate was diluted in 2.4 volumes of the same isolating medium. Duplicates from each sample were prepared. The diluted myofibril suspension was stirred for 10 s and dispensed into a cuvette. The absorbance of this suspension was measured immediately at 540 nm using a Spectronic 20 Bausch and Lomb Colorimeter. The average absorbance measurements were multiplied by a factor of 200 to give an MFI for that particular steak.

### 2.8 Sarcomere length

Sarcomere length was determined as described by Culler et al. (1978) on diluted myofibril suspensions from the short MFI method. Protein concentrations (0.5 ± 0.05 mg/ml) were determined by the modified biuret method of Robson et al. (1968). A drop of the myofibril suspension was placed on a glass slide. The microscope slide was partitioned into imaginary quadrants and 24 myofibrils per quadrant were measured using a Vicker's image splitter attached to a Zeiss photomicroscope. The photomicroscope was adapted with a Neofluar 40× phase objective. Average sarcomere lengths in micrometer were determined using the following equation:

$$\frac{\mathrm{Sum}\;\text{of lengths measured}/\mathrm{sum}\;\text{of numbers of sarcomeres}}{\left(1.33=\text{correction factor}\right)}.$$

### 2.9 Statistical analysis

Data were analyzed initially using the GLM procedure of SAS (SAS 1985, Version 8.01) conducting an analysis of variance. Least square means, correlation coefficients, and standard error of means were generated for all traits of interest. The least significant difference tests were carried out to detect the effect of pH on palatability, sarcomere length, MFI, and shear force measures. Mean differences among groups were compared using Tukey's post hoc test. All tests are reported as significant using *α* = 0.05. Statistical analysis did not include sensory data, MFI, and WBSF from the single PSE sample.

## 3 RESULTS AND DISCUSSION

### 3.1 Carcass traits, color, and pH

Carcasses used in the present study were classified on the basis of visual appearance (pale, soft, exudative [PSE] normal or dark color [DFD]), colorimetric, and pH measurement. LTL samples were divided into four different groups; low/normal pH (5.5), PSE (pH 5.5), intermediate pH (6.0–6.1), and high pH (6.4–6.9). This classification has been used by Wu et al. (2014), Ponnampalum et al. (2017), and Yu and Lee (1986). Table 1 shows the distribution of carcasses by weight, maturity, and marbling score. The groups were of similar maturity but carcasses within each marbling category averaged as ‘Slight’ in terms of degrees of marbling but higher scores were evident at the higher pH.

**TABLE 1:** Selected carcass traits^a^ at 24 h postmortem from carcasses varying in pH.

Trait	pH			
	5.5 (Normal)	5.5 (PSE)	6.0–6.1	6.4–6.9
No of Carcasses	15 (9 bulls + 6 steers)	1 (bull)	7 (4 bulls + 3 steers)	7 (4 bulls + 3 steers)
Carcass wt (kg)	316.8 ± 11.3	321.8	310.1 ± 10.4	333.2 ± 10.1
Maturity	A	A	A	A
Marbling score^b^	941 ± 25	963	956 ± 30	977 ± 30
*L* (lightness)	45.2 ± 0.6^a^	55.9 ± 0.3^b^	27.2 ± 0.3^c^	32.4 ± 1.0^d^
*a* (redness)	9.5 ± 0.3^a^	5.2 ± 0.2^b^	6.7 ± 0.7^c^	6.1 ± 1.1^c^
*b* (yellowness)	12.9 ± 0.6^a^	16.1 ± 0.1^b^	7.7 ± 0.4^c^	8.4 ± 0.3^c^

*Note*: ^a–d^Means bearing the same superscripts are not significantly different (*p* < .05).

Abbreviation: PSE, pale soft exudative.

^a^ Means ± standard deviations.

^b^ Slight^0^ = 900, Small^0^ = 1000.

*L* (lightness) scores for DFD steaks were significantly (*p* < .05) less than normal pH cuts, while PSE exhibited significantly higher values. Redness (*a*) values did not differ significantly for DFD but differed when compared with normal and PSE meat. Normal pH steaks showed higher *b* (yellowness) values (*p* < .05) in comparison with the DFD group. PSE meat was significantly higher than the other groups. These results concur with findings from Barón et al. (2021) as well as Livisay et al. (1996). Steaks with a high or intermediate pH will have a lower reflectance of light due to their greater water‐holding capacity, providing a closed structure impeding oxygen diffusion and allowing for greater light absorption and reduced reflectance (Hughes et al., 2017). The aforementioned authors also found higher myoglobin content and deoxymyoglobin concentrations when compared to other muscle pHs.

### 3.2 Shear force, MFI, and sensory attributes

Significantly high correlations between WBSF and sensory tenderness scores have been obtained by a number of authors (Lee et al., 2022; Silva et al., 2017). Results of physical and palatability attributes for LTL muscles based on pH are given in Table 2. A significant difference (*p* < .05) was observed for sensory tenderness between all the pH groups. Intermediate pH meat showed an increase in toughness, while high pH beef was the most tender. Studies have reported the effect of pH on the tenderness of beef (Aalhus et al., 2007; Grayson et al., 2016 and Wu et al., 2014) and their results are consistent with the findings given here. A curvilinear relationship was obtained for sensory tenderness and WBSF values by Boudjellal et al. (2008), and once again, our findings concur with previous work. Pulford et al. (2009) as well as Lomiwes et al. (2014) hypothesized a shielding effect by high levels of αβ‐crystallin toward myofibrillar proteins as a response to protein denaturation at intermediate pH range (5.7 < pH < 6.3) preventing endopeptidase degradation while at pH <5.7, denaturation of proteins renders the myofibrillar structure more susceptible to proteolysis. When comparing bulls and steers, no differences were found between shear forces at similar pH for samples of LT muscle by Purchas (1990).

**TABLE 2:** The least square means palatability, shear force, MFI, and sarcomere length values for *longissimus thoracis et lumborum* muscle at different pH following 14 days of aging.

Trait	pH			SEM
	5.5	6.0–6.1	6.4–6.9	
Tenderness	6.7^x^	5.5^y^	7.4^z^	0.16
Connective tissue	6.8^xy^	6.7^x^	7.1^y^	0.10
Juiciness	6.6^xy^	6.4^x^	7.0^y^	0.13
Flavor intensity	6.7^xy^	6.5^x^	7.0^y^	0.13
Flavor	6.7^x^	4.2^y^	3.9^y^	0.16
Myofibrillar force (kg/cm^2^)	2.6^x^	2.7^x^	1.6^y^	0.21
Residual force (kg/cm^2^)	2.5^x^	2.8^x^	1.8^y^	0.22
Sarcomere length (μm)	2.10^y^	2.01^x^	2.10^y^	0.01
MFI	51.6^x^	48.0^x^	60.0^y^	3.80

*Note*: ^x–z^Means bearing the same superscripts are not significantly different (*p* < .05).

Abbreviations: MFI, myofibril fragmentation index; SEM, standard error of the mean.

Connective tissue, juiciness, and flavor intensity differed significantly between high pH and intermediate pH ranges. No significant difference existed between normal pH samples and those at other pHs for these same sensory parameters. Juiciness scores tended to be reduced in the intermediate pH range and improved on either side of this. These findings were confirmed by Holdstock et al. (2014) previously. Purchas (1990) reported that expressed juice decreased linearly with an increase in pH but was not different after adjusting for pH. Flavor scores for normal pH and dark‐cutting beef were found to be significantly different (*p* < .05), although no significant difference was detected amongst dark cutters themselves. It is conceivable that a breakdown product from the 30 kD (see below) may be involved in the flavor changes and perception of dark‐cutting meat. Researchers (Jeremiah et al., 1988) found that steaks from steers under minimum preslaughter stress were rated higher than their counterparts from bulls when under normal preslaughter stress. This would suggest that steer steaks contain appropriate flavor character notes.

Myofibril fragmentation indices reported in Table 2 also showed a significant and dramatic effect at the high pH range with greater values when compared to intermediate and normal pH, myofibril fracture at high pH is almost instantaneous. Samples at this high pH were significantly different from all samples between pH 5.5 and pH 6.1. The higher shear force values at intermediate pH as well as the lower shear force values of high pH steaks corroborate the findings of Prieto et al. (2018). Yu and Lee (1986) found *longissimus* muscle at intermediate pH (5.8–6.3) to have higher shear force values than at either low (5.8) or high (6.3) pH. Data presented by Dransfield (1981) showed minimum tenderness existed at a pH of approximately 6.1. Purchas (1990) attributed increased shear force values at pH 6.1, at least partly, to sarcomere length, although Yu and Lee produced evidence to the contrary with no significant differences being found for sarcomere length at all pH groups. Our measurements of sarcomere length were larger at normal pH and pH 6.7–6.9 were significantly different (*p* < .05) from those at pH 6.0–6.1.

Data in Table 3 are correlation coefficients among various physical, chemical, and sensory characteristics of *LTL* muscle from dark‐cutting beef. The most significant relationships were among MFI, sensory tenderness, myofibrillar force measurement, and residual force measurement. Correlations between MFI and the aforementioned parameters were 0.73, −0.77, and −0.76, respectively. Sensory tenderness scores were significantly correlated to myofibrillar force (−0.80) and residual force (−0.69). No significant correlations were computed between sarcomere length and other variables. However, Battaglia et al. (2020) found that sarcomere length and shear force were both correlated with tenderness, using laser diffraction and microscopy to measure sarcomere length. More recently, Briggs et al. (2021) found through regression analyses that the abundance of oxidative stress protein deglycase after 14 days of aging is a predictor of WBSF (*p* < .001), MFI (*p* = .02), and sensory panel tenderness (*p* < .001). The abundance of heat shock protein β1 after 14 days of aging was also a predictor of MFI (*p* = .03).

**TABLE 3:** Correlation coefficients among physical, chemical, and sensory characteristics of *longissimus thoracis et lumborum* muscle of dark‐cutting beef following 14 days of aging.

	MFI	Sensory tenderness	Myofibrillar force	Residual force
Sensory tenderness	0.73**			
Myofibrillar force	−0.77**	0.80**		
Residual force	−0.76**	−0.69**	0.80**	
Sarcomere length	0.26	0.34	−0.34	−0.39

Abbreviation: MFI, myofibril fragmentation index.

** *p* < .01.

Marbling, MFI, and sensory tenderness were significantly correlated, although they were of low magnitude.

### 3.3 Myofibrillar protein degradation patterns

**FIGURE 1:**
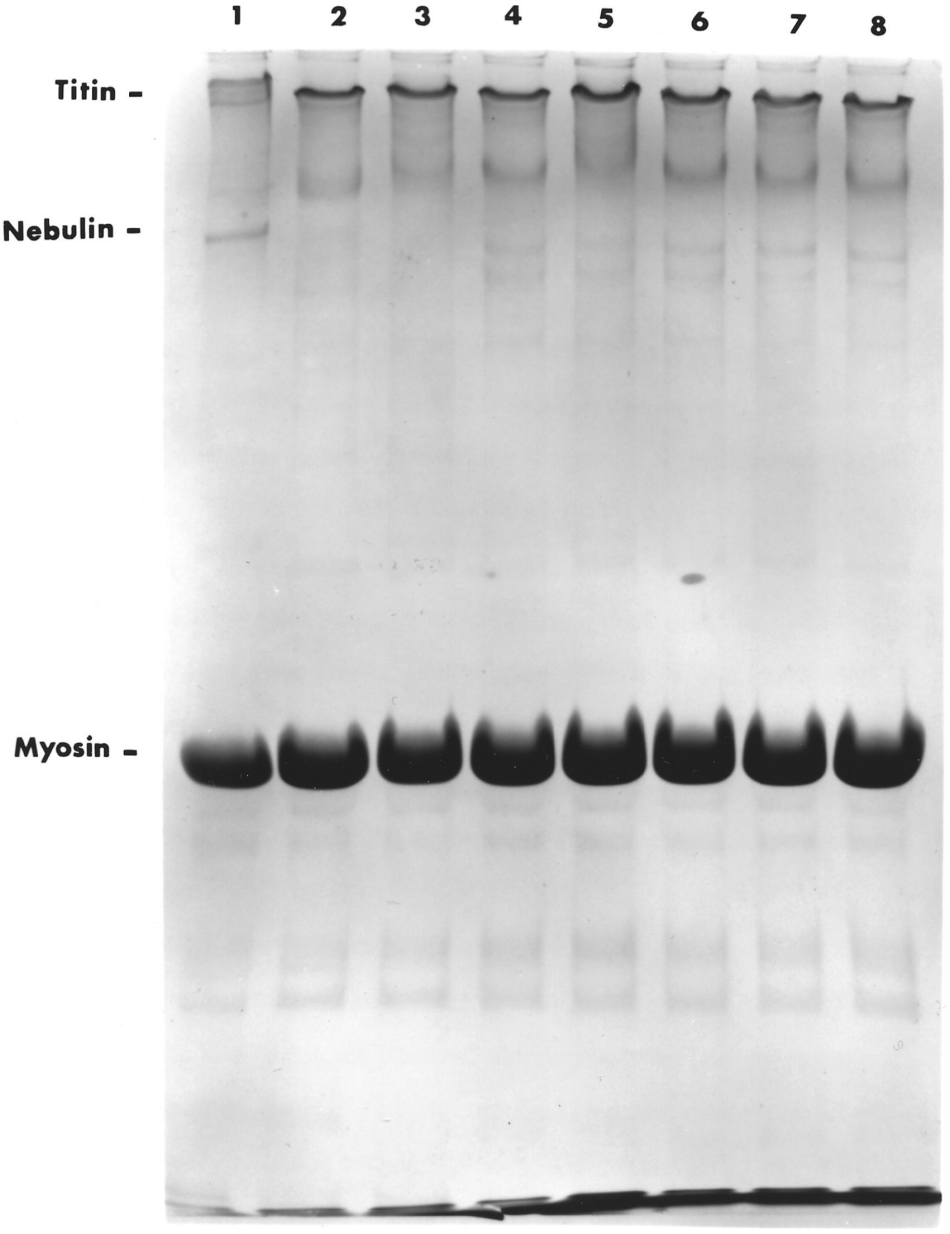
Five‐percent polyacrylamide gel electrophoretograms of myofibrillar/cytoskeletal proteins of bovine *longissimus thoracis et lumborum* muscle following 14‐day aging. Lane 1 represents beef sample at 3 days postmortem; lane 2, pH 5.5 (normal beef); lane 3, pH 5.5 (pale soft exudative); lane 4, pH 6.0; lane 5, pH 6.1; lane 6, pH 6.4; lane 7, pH 6.7; and lane 8, pH 6.9, respectively. Protein concentrations in each lane were 120 μg.

**FIGURE 2:**
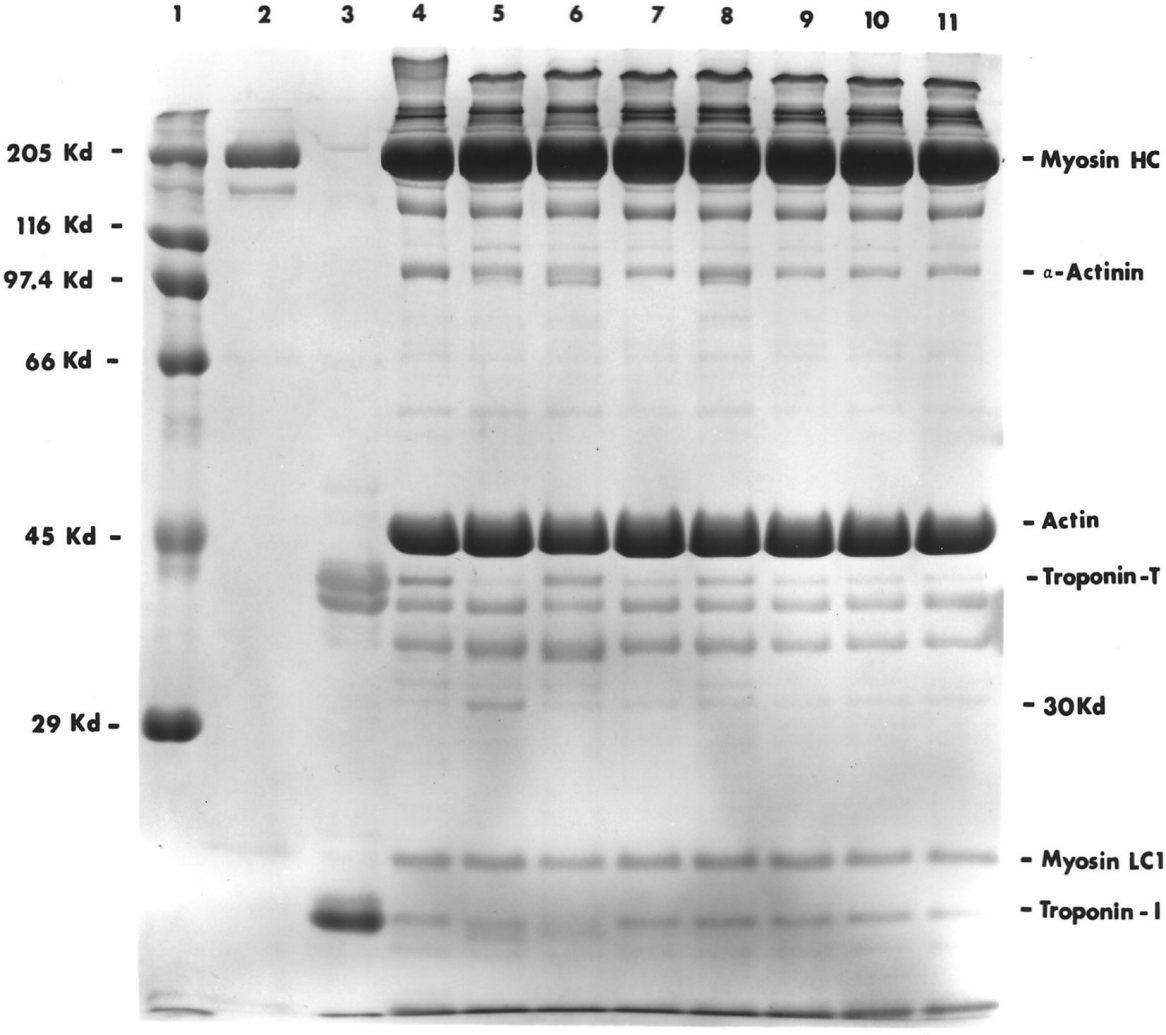
Twelve‐percent polyacrylamide gel electrophoretograms, containing a 5% stacking gel of myofibrillar/cytoskeletal proteins of bovine *longissimus thoracis et lumborum* muscle following 14‐day aging. Lane 1 represents molecular weight standards; lane 2, myosin standard; lane 3, troponin‐T standard; lane 4, beef sample at 3 days postmortem; lane 5, pH 5.5 (normal beef); lane 6, pH 5.5 (pale soft exudative); lane 7, pH 6.0; lane 8, pH 6.1; lane 9, pH 6.4; lane 10, pH 6.7; and lane 11, pH 6.9, respectively. Protein concentrations in lanes 1–3 were 60 μg while lanes 5–11 were 120 μg.

In the articles by Lomiwes et al. (2014) and Gagaoua et al. (2020), discussion regarding the development of meat tenderness is explained overwhelmingly due to pH and enzymatic breakdown. The aging of meat allows for the proteolytic breakdown of myofibrillar/cytoskeletal proteins, disrupting the integrity of muscle cells (Bhat et al., 2018). SDS‐PAGE patterns of purified myofibrils from *LTL* muscles of various pHs following a 14‐day aging period are shown in Figures 1 and 2. Qualitative visual analysis of these myofibrillar proteins and fragments has been extensively reported previously using SDS‐PAGE (Huff‐Lonergan et al., 1995; MacBride & Parrish, 1977; Pommier et al., 1987). In this light, we provide evidence of both low‐ and high‐molecular‐weight protein degradation patterns.

Figure 1 depicts SDS‐5% gels used to separate the high‐molecular‐weight proteins while SDS‐12% was used for greater resolution of the lower molecular weight protein spectrum. Resolution of the high‐molecular‐weight proteins revealed the total degradation of nebulin and that titin was present in the T2 form. Titin and nebulin are gigantic, longitudinally running proteins of the myofibril anchored at one of their ends of the Z‐line (Huff‐Lonergan et al., 2010). Lane 1 shows a tough steer at 3 days postmortem with distinct Titin (T1 and T2) and nebulin bands. It has been described by Fritz and Greaser (1991) that degradation of Titin from T1 to T2 and not the absolute amount of titin in postmortem muscle contributes to postmortem tenderization.

Cruzen (2013) reported that two of the most well‐known isoforms of calpain (μ‐calpain and m‐calpain) cleave the same myofibrillar proteins that are degraded during postmortem aging without degrading a great deal of actin or myosin. Titin is a known substrate for calpain (Zeece et al., 1986) and nebulin degradation (Huff‐Lonergan et al., 1995). Calpains act rapidly on the intermediate filaments, desmin, (encircling the Z‐line periphery and connecting myofibrils) (O'Shea et al., 1979), and synemin, a protein co‐localized with desmin at the Z‐line periphery (Robson et al., 1991). It is possible that titin, nebulin, desmin, and synemin play a greater role in tenderness than any of the other myofibrillar proteins during aging at high pH.

Muscle samples representing pH 6.0 through 6.9 also revealed a “doublet” of slightly lower molecular weight than nebulin even though pH 6.1 showed this band to be of lesser intensity. Chang et al. (2019) found that calpains are maximally active between pH 6.5 and 8.5. However, Dransfield (1992) claims from modeling postmortem tenderization that as the pH of muscle falls to about 6.1, calpain I is activated with the increase in calcium ion concentration to about 10^−4^ M. It is evident that due to the lack of lysosomal activity and neutral proteases, samples at the intermediate pH range were susceptible to limited degradation. This conclusion is supported by Yu and Lee (1986) when they examined samples of longissimus muscle between pH 5.8 and 6.3 by electron microscopy and found limited degradation of Z‐lines, thick and thin filaments. Furthermore, findings by Wu et al. (2014) stated that the slowest rate of degradation was evident at intermediate pH.

Olson and Parrish (1977) demonstrated marked and specific proteolysis in bovine muscle undergoing postmortem storage at normal pH. They found the simultaneous disappearance of troponin‐T and the concomitant appearance of a 30 kDa component to be indicative of calcium‐activated protease activity. This intriguing finding was also shown to occur only in tender and not tough bovine longissimus (MacBride & Parrish, 1977). This present study revealed a striking finding amongst the dark cutters of high pH (6.7–6.9). It is apparent (Figure 2, lanes 9–11) that troponin‐T was almost completely degraded while traces of the 30 kD component were barely visible. This could be due to further degradation of the 30 kD at high pH which has never been described before. Control samples at pH 5.5 showed marked intensities of 30 kD, whereas the tough steer (lane 4) and PSE sample (lane 6) showed negligible amounts of this product. It is evident that PSE beef (pH 5.5) had a very similar protein degradation pattern to the tough steer, with little breakdown of troponin‐T to 30 kD except that troponin‐l appears to have disintegrated. Decreased activation of μ‐calpain (Bee et al., 2007) may be part of the reason for the lack of myofibrillar degradation patterns seen in this current study. To the best of our knowledge, there is no publication showing such electrophoretic proof of myofibrillar patterns for normal, PSE, and DFD beef comparisons.

### 3.4 Implications

The implications of such findings lend to a better appreciation of the mechanism for the proteolytic activity taking place at varying pHs. Thus, we propose that the activation of μ‐calpain at near neutral pH results in the rapid degradation of titin and nebulin as well as other myofibrillar/cytoskeletal proteins such as desmin, filamin, synemin, and troponin‐T. At intermediate pH, a reduction in μ‐calpain activity as well as the competitive inhibition by αβ‐crystallin and calpastatin slow down the proteolytic breakdown. In contrast, at low pH, cathepsins dominate enzymic breakdown. Of course the above is simplistic, proteolysis during postmortem aging is a complex process with many aspects involving a concerted array of measures. In addition, stress‐related syndromes confound the course of events, where aging of PSE beef results also brings into play further changes such as the degradation of troponin‐I, and in DFD, the breakdown products of 30 kD. The above‐mentioned proteins constitute important fractions of myofibrillar proteins. However, there are also other myofibrillar proteins that certainly contribute to the overall tenderness properties of beef and their role should be taken into context.

## 4 CONCLUSIONS

SDS‐PAGE provided unique fingerprints of protein degradation patterns from LTL muscle aged for 14 days at various pHs. The results shed new light on our understanding of the myofibrillar proteolysis occurring during aging. Despite the breakdown of Titin T1 to T2 and proteolysis of nebulin, troponin‐T breakdown to 30 kD was incomplete in PSE beef as well as DFD beef at pH 6.0–6.1. At the high pH extreme, troponin‐T had almost disappeared and the 30 kD was not present. This intriguing finding adds further credence to the complex tenderness conundrum attributed to myofibrillar breakdown and structural integrity. The tenderness experienced at high pH was corroborated by MFI, larger sarcomere lengths, and shear force measures. PSE beef revealed the breakdown of troponin‐I suggesting a different proteolytic mechanism during postmortem aging. Further research work is required to elucidate the mechanism by which pH affects the tenderness properties of beef.

## CONFLICT OF INTEREST

The authors declare that they do not have any conflict of interest.

## Data Availability

The data that support the findings of this study are available from the corresponding author upon reasonable request.
